# Supplementary material for: A survey of authors publishing in four megajournals
Source: PeerJ. 2014 Apr 22;2:e365. doi: 10.7717/peerj.365 (PMC4006221; doi:10.7717/peerj.365)
Supplement: Supplemental Information 1 [file peerj-02-365-s001.docx]

| **Author country affiliation** | | | | | | | |
| --- | --- | --- | --- | --- | --- | --- | --- |
|  | | | Journal | | | | Total |
|  |  |  | BMJ | PeerJ | PLoS | Sage |  |
| Country | Not Marked | Count | 0 | 2 | 3 | 1 | 6 |
|  |  | Percent | 0.0% | 2.1% | 1.1% | .9% | .9% |
|  | Argentina | Count | 0 | 0 | 1 | 0 | 1 |
|  |  | Percent | 0.0% | 0.0% | .4% | 0.0% | .2% |
|  | Austria | Count | 1 | 0 | 0 | 0 | 1 |
|  |  | Percent | .5% | 0.0% | 0.0% | 0.0% | .2% |
|  | Australia | Count | 23 | 4 | 9 | 5 | 41 |
|  |  | Percent | 12.2% | 4.2% | 3.3% | 4.6% | 6.2% |
|  | Belgium | Count | 0 | 1 | 1 | 0 | 2 |
|  |  | Percent | 0.0% | 1.1% | .4% | 0.0% | .3% |
|  | Burkina Faso | Count | 0 | 0 | 1 | 0 | 1 |
|  |  | Percent | 0.0% | 0.0% | .4% | 0.0% | .2% |
|  | Brazil | Count | 4 | 1 | 9 | 1 | 15 |
|  |  | Percent | 2.1% | 1.1% | 3.3% | .9% | 2.3% |
|  | Canada | Count | 11 | 9 | 11 | 10 | 41 |
|  |  | Percent | 5.8% | 9.5% | 4.0% | 9.2% | 6.2% |
|  | Switzerland | Count | 0 | 0 | 2 | 1 | 3 |
|  |  | Percent | 0.0% | 0.0% | .7% | .9% | .5% |
|  | China | Count | 4 | 0 | 44 | 0 | 48 |
|  |  | Percent | 2.1% | 0.0% | 16.2% | 0.0% | 7.2% |
|  | Colombia | Count | 0 | 0 | 1 | 0 | 1 |
|  |  | Percent | 0.0% | 0.0% | .4% | 0.0% | .2% |
|  | Costa Rica | Count | 0 | 1 | 0 | 0 | 1 |
|  |  | Percent | 0.0% | 1.1% | 0.0% | 0.0% | .2% |
|  | Cape Verde | Count | 1 | 0 | 0 | 0 | 1 |
|  |  | Percent | .5% | 0.0% | 0.0% | 0.0% | .2% |
|  | Czech Republic | Count | 0 | 0 | 1 | 0 | 1 |
|  |  | Percent | 0.0% | 0.0% | .4% | 0.0% | .2% |
|  | Germany | Count | 7 | 3 | 17 | 1 | 28 |
|  |  | Percent | 3.7% | 3.2% | 6.3% | .9% | 4.2% |
|  | Denmark | Count | 10 | 1 | 3 | 0 | 14 |
|  |  | Percent | 5.3% | 1.1% | 1.1% | 0.0% | 2.1% |
|  | Spain | Count | 0 | 0 | 4 | 2 | 6 |
|  |  | Percent | 0.0% | 0.0% | 1.5% | 1.8% | .9% |
|  | Ethiopia | Count | 1 | 0 | 1 | 0 | 2 |
|  |  | Percent | .5% | 0.0% | .4% | 0.0% | .3% |
|  | Finland | Count | 3 | 0 | 0 | 2 | 5 |
|  |  | Percent | 1.6% | 0.0% | 0.0% | 1.8% | .8% |
|  | France | Count | 5 | 1 | 7 | 1 | 14 |
|  |  | Percent | 2.6% | 1.1% | 2.6% | .9% | 2.1% |
|  | United Kingdom | Count | 51 | 7 | 18 | 8 | 84 |
|  |  | Percent | 27.0% | 7.4% | 6.6% | 7.3% | 12.6% |
|  | Guadeloupe | Count | 0 | 0 | 1 | 0 | 1 |
|  |  | Percent | 0.0% | 0.0% | .4% | 0.0% | .2% |
|  | Greece | Count | 0 | 2 | 1 | 1 | 4 |
|  |  | Percent | 0.0% | 2.1% | .4% | .9% | .6% |
|  | Indonesia | Count | 1 | 0 | 1 | 0 | 2 |
|  |  | Percent | .5% | 0.0% | .4% | 0.0% | .3% |
|  | Ireland | Count | 4 | 0 | 0 | 2 | 6 |
|  |  | Percent | 2.1% | 0.0% | 0.0% | 1.8% | .9% |
|  | Israel | Count | 0 | 1 | 1 | 2 | 4 |
|  |  | Percent | 0.0% | 1.1% | .4% | 1.8% | .6% |
|  | India | Count | 2 | 1 | 1 | 3 | 7 |
|  |  | Percent | 1.1% | 1.1% | .4% | 2.8% | 1.1% |
|  | Iran, Islamic Republic of | Count | 2 | 0 | 0 | 2 | 4 |
|  |  | Percent | 1.1% | 0.0% | 0.0% | 1.8% | .6% |
|  | Iceland | Count | 1 | 0 | 0 | 0 | 1 |
|  |  | Percent | .5% | 0.0% | 0.0% | 0.0% | .2% |
|  | Italy | Count | 8 | 1 | 11 | 1 | 21 |
|  |  | Percent | 4.2% | 1.1% | 4.0% | .9% | 3.2% |
|  | Japan | Count | 10 | 2 | 12 | 1 | 25 |
|  |  | Percent | 5.3% | 2.1% | 4.4% | .9% | 3.8% |
|  | Korea, Republic of | Count | 0 | 0 | 7 | 0 | 7 |
|  |  | Percent | 0.0% | 0.0% | 2.6% | 0.0% | 1.1% |
|  | Kuwait | Count | 0 | 0 | 0 | 1 | 1 |
|  |  | Percent | 0.0% | 0.0% | 0.0% | .9% | .2% |
|  | Lebanon | Count | 1 | 0 | 0 | 0 | 1 |
|  |  | Percent | .5% | 0.0% | 0.0% | 0.0% | .2% |
|  | Malta | Count | 0 | 0 | 0 | 1 | 1 |
|  |  | Percent | 0.0% | 0.0% | 0.0% | .9% | .2% |
|  | Mexico | Count | 0 | 1 | 0 | 0 | 1 |
|  |  | Percent | 0.0% | 1.1% | 0.0% | 0.0% | .2% |
|  | Malaysia | Count | 1 | 1 | 2 | 1 | 5 |
|  |  | Percent | .5% | 1.1% | .7% | .9% | .8% |
|  | Nigeria | Count | 0 | 0 | 0 | 2 | 2 |
|  |  | Percent | 0.0% | 0.0% | 0.0% | 1.8% | .3% |
|  | Netherlands | Count | 8 | 3 | 11 | 0 | 22 |
|  |  | Percent | 4.2% | 3.2% | 4.0% | 0.0% | 3.3% |
|  | Norway | Count | 3 | 1 | 5 | 3 | 12 |
|  |  | Percent | 1.6% | 1.1% | 1.8% | 2.8% | 1.8% |
|  | Nepal | Count | 2 | 0 | 0 | 0 | 2 |
|  |  | Percent | 1.1% | 0.0% | 0.0% | 0.0% | .3% |
|  | New Zealand | Count | 1 | 1 | 1 | 1 | 4 |
|  |  | Percent | .5% | 1.1% | .4% | .9% | .6% |
|  | Philippines | Count | 0 | 0 | 0 | 1 | 1 |
|  |  | Percent | 0.0% | 0.0% | 0.0% | .9% | .2% |
|  | Poland | Count | 0 | 0 | 2 | 0 | 2 |
|  |  | Percent | 0.0% | 0.0% | .7% | 0.0% | .3% |
|  | Puerto Rico | Count | 0 | 1 | 0 | 0 | 1 |
|  |  | Percent | 0.0% | 1.1% | 0.0% | 0.0% | .2% |
|  | Portugal | Count | 0 | 1 | 0 | 2 | 3 |
|  |  | Percent | 0.0% | 1.1% | 0.0% | 1.8% | .5% |
|  | Qatar | Count | 1 | 0 | 0 | 0 | 1 |
|  |  | Percent | .5% | 0.0% | 0.0% | 0.0% | .2% |
|  | Romania | Count | 0 | 0 | 1 | 0 | 1 |
|  |  | Percent | 0.0% | 0.0% | .4% | 0.0% | .2% |
|  | Saudi Arabia | Count | 0 | 0 | 0 | 1 | 1 |
|  |  | Percent | 0.0% | 0.0% | 0.0% | .9% | .2% |
|  | Sweden | Count | 5 | 3 | 5 | 3 | 16 |
|  |  | Percent | 2.6% | 3.2% | 1.8% | 2.8% | 2.4% |
|  | Singapore | Count | 0 | 0 | 1 | 0 | 1 |
|  |  | Percent | 0.0% | 0.0% | .4% | 0.0% | .2% |
|  | Thailand | Count | 0 | 1 | 0 | 2 | 3 |
|  |  | Percent | 0.0% | 1.1% | 0.0% | 1.8% | .5% |
|  | Tunisia | Count | 0 | 0 | 0 | 1 | 1 |
|  |  | Percent | 0.0% | 0.0% | 0.0% | .9% | .2% |
|  | Turkey | Count | 0 | 0 | 1 | 0 | 1 |
|  |  | Percent | 0.0% | 0.0% | .4% | 0.0% | .2% |
|  | Taiwan, Province of China | Count | 0 | 0 | 7 | 0 | 7 |
|  |  | Percent | 0.0% | 0.0% | 2.6% | 0.0% | 1.1% |
|  | Tanzania, United Republic of | Count | 0 | 0 | 1 | 0 | 1 |
|  |  | Percent | 0.0% | 0.0% | .4% | 0.0% | .2% |
|  | Uganda | Count | 1 | 0 | 0 | 0 | 1 |
|  |  | Percent | .5% | 0.0% | 0.0% | 0.0% | .2% |
|  | United States | Count | 16 | 44 | 63 | 46 | 169 |
|  |  | Percent | 8.5% | 46.3% | 23.2% | 42.2% | 25.4% |
|  | Viet Nam | Count | 0 | 0 | 1 | 0 | 1 |
|  |  | Percent | 0.0% | 0.0% | .4% | 0.0% | .2% |
|  | South Africa | Count | 1 | 1 | 3 | 0 | 5 |
|  |  | Percent | .5% | 1.1% | 1.1% | 0.0% | .8% |
| Total | | Count | 189 | 95 | 272 | 109 | 665 |
|  |  | Percent | 100.0% | 100.0% | 100.0% | 100.0% | 100.0% |
