# Supplementary material for: A survey of authors publishing in four megajournals
Source: PeerJ. 2014 Apr 22;2:e365. doi: 10.7717/peerj.365 (PMC4006221; doi:10.7717/peerj.365)
Supplement: Supplemental Information 2 [file peerj-02-365-s002.docx]

**For those authors who have academic positions**

|  | | | | | | |
| --- | --- | --- | --- | --- | --- | --- |
| **If you have an academic position, tenure status** | | | | | | |
|  | | Journal | | | | Total |
|  |  | BMJ | PeerJ | PLoS | Sage |  |
| Non-tenure stream | Count | 111 | 48 | 163 | 62 | 384 |
|  | % within Journal | 58.7% | 50.5% | 59.9% | 56.9% | 57.7% |
| Not Tenured | Count | 38 | 17 | 41 | 14 | 110 |
|  | % within Journal | 20.1% | 17.9% | 15.1% | 12.8% | 16.5% |
| Tenured | Count | 40 | 30 | 68 | 33 | 171 |
|  | % within Journal | 21.2% | 31.6% | 25.0% | 30.3% | 25.7% |
|  | Count | 189 | 95 | 272 | 109 | 665 |
|  | % within Journal | 100.0% | 100.0% | 100.0% | 100.0% | 100.0% |

|  |  |  |  |  |  |  |
| --- | --- | --- | --- | --- | --- | --- |
|  |  |  |  |  |  |  |
| **IIf you have an academic position, rank** | | | | | | |
|  | | Journal | | | | Total |
|  |  | BMJ | PeerJ | PLoS | Sage |  |
| Not relevant | Count | 108 | 42 | 133 | 53 | 336 |
|  | % within Journal | 57.1% | 44.2% | 48.9% | 48.6% | 50.5% |
| Assistant Professor | Count | 25 | 19 | 35 | 22 | 101 |
|  | % within Journal | 13.2% | 20.0% | 12.9% | 20.2% | 15.2% |
| Associate Professor | Count | 23 | 20 | 42 | 13 | 98 |
|  | % within Journal | 12.2% | 21.1% | 15.4% | 11.9% | 14.7% |
| Full Professor | Count | 33 | 14 | 62 | 21 | 130 |
|  | % within Journal | 17.5% | 14.7% | 22.8% | 19.3% | 19.5% |
|  | Count | 189 | 95 | 272 | 109 | 665 |
|  | % within Journal | 100.0% | 100.0% | 100.0% | 100.0% | 100.0% |
|  |  |  |  |  |  |  |
